# Higher *P*-Wave Dispersion in Migraine Patients with Higher Number of Attacks

**DOI:** 10.1100/2012/791460

**Published:** 2012-05-01

**Authors:** A. Koçer, M. Eryılmaz, H. Tutkan, N. Ercan, Z. S. Küçükbayrak

**Affiliations:** ^1^Neurology Department, Medical Faculty, Bezmialem Vakif University, Istanbul, Turkey; ^2^Neurology Department, Medical Faculty, Düzce University, Düzce, Turkey; ^3^Neurology Department, Sakarya State Hospital, Sakarya, Turkey; ^4^Physiology Department, Medical Faculty, Düzce University, Düzce, Turkey

## Abstract

*Objective and Aim*. An imbalance of the sympathetic system may explain many of the clinical manifestations of the migraine. We aimed to evaluate *P*-waves as a reveal of sympathetic system function in migraine patients and healthy controls. *Materials and Methods*. Thirty-five episodic type of migraine patients (complained of migraine during 5 years or more, BMI < 30 kg/m^2^) and 30 controls were included in our study. We measured *P*-wave durations (minimum, maximum, and dispersion) from 12-lead ECG recording during pain-free periods. ECGs were transferred to a personal computer via a scanner and then used for magnification of x400 by Adobe Photoshop software. *Results*. *P*-wave durations were found to be similar between migraine patients and controls. Although *P* WD (*P*-wave dispersion) was similar, the mean value was higher in migraine subjects. *P* WD was positively correlated with *P* max (*P* < 0.01). Attacks number per month and male gender were the factors related to the *P* WD (*P* < 0.01). *Conclusions*. Many previous studies suggested that increased sympathetic activity may cause an increase in *P* WD. We found that *P* WD of migraine patients was higher than controls, and *P* WD was related to attacks number per month and male gender. Further studies are needed to explain the chronic effects of migraine.

## 1. Introduction

It is widely accepted that symptoms of migraine are related to the involvement of the autonomic nervous system, and dysfunction may affect atrial and ventricular repolarization. The sympathetic nervous system is known to play an important role in the genesis of ventricular arrhythmias [1]. QT interval changes and *P*-wave changes may be predictors of atrial and ventricular arrhythmias. The most significant one is prolonged QT interval, which is a predictor of ventricular arrhythmias [2]. In addition, *P*-wave dispersion which is a predictor of atrial fibrillation (AF) is defined as the difference between maximum and minimum *P*-wave duration and has been associated with inhomogeneous and discontinuous propagation of sinus impulses [3, 4]. It has been shown that increased sympathetic activity caused a significant elevation in *P*-wave dispersion [5]. It has also been reported that there was an association between the autonomic nervous system and atrial fibrillation [6, 7].

Although the previous reports reflected atrial and ventricular repolarization abnormalities that were affected by disturbed ANS (sympathetic and/or parasympathetic nervous system dysfunction) during migraine attacks, the association between atrial fibrillation and migraine is limited to case reports only [8–12]. Duru et al. reported that maximum *P*-wave duration (*P* max) and *P*-wave disperson (*P* WD) were found higher during migraine attacks than during pain-free periods [13]. However, *P* WD was not reported during pain-free period which might be a shower of damage related to attacks until now in comparison to healthy controls. In this study, we tried to find whether the patients with migraine may go under the risk of atrial and ventricular arrhythmias or not. For this reason, we undertook evaluation of *P*-wave dispersion as a sign of autonomic dysfunction in patients with well-defined migraine during headache-free period and compared to normal healthy controls.

## 2. Methods

Thirty-five episodic type of migraine patients (complained of migraine during 5 years or more, BMI < 30 kg/m^2^), and age and sex-matched 30 healthy controls were included in our study.

The diagnosis of migraine was made using criteria of the International Headache Society [14]. Thirty-five subjects with migraine were evaluated during the pain-free period; 14 with migraine with aura (MWA) and 21 with migraine without aura (MWOA). We confined the study to women aged 20 to 45 years who had suffered from migraine for more than 1 year and had at least one migraine attack per month. The presence of other pain syndromes (e.g., chronic low back pain or chronic tension-type headache), systemic disease (e.g., diabetes mellitus), and disorders that could affect the autonomic nervous system were exclusionary. Except for mild analgesics, all drugs were withdrawn 5 days before the testing, and no drug, including caffeine, or cigarettes, was allowed on the day of testing. The control group (*n* = *l*6) consisted of age and sex-matched persons who were free of migraine, other chronic pain syndromes, systemic diseases, or disorders that could affect the autonomic nervous system. These subjects were not on any medication. The study protocol was approved by the Institutional Review Board.

Autonomic tests were performed in the headache-free period. None of the subjects reported headache for at least 72 h before and after testing. Subjects were instructed to abstain from caffeine-containing beverages and, in the adolescents, from nicotine and alcohol for at least 24 h before the test. The examinations took place between 14.00 h and 17.00 h to avoid diurnal variation and were performed in a warm room (24°C). The subjects were asked to lie down and not to move during recording. The ECG recording was performed. Heart rate, *P* max and minimum *P*-wave duration (*P* min), and *P* WD were measured from 12-lead ECG recording during pain-free periods. The difference between the maximum and minimum *P*-wave duration was defined as *P* WD. ECGs were transferred to a personal computer via a scanner and then used for magnification of x400 by Adobe Photoshop software.

Intra- and interobserver coefficients of variation (standard deviation [SD] of differences between 2 observations divided by the mean value and expressed in percent) were found as 3.7% and 3.8% for *P*-wave dispersion. Intra and interobserver coefficients of variation were found to be less than 5%. All data were presented as mean value ± SD. Comparison of clinical variables between 2 groups was performed with paired Student *t*-test for numeric variables and chi-square test for categorical data. A *P* value < 0.05 was considered to be statistically significant. The SPSS version 11.0 package was used in statistical analysis.

## 3. Results

Sociodemographical and clinical findings and *P*-wave values were summarized in Table 1. Ten patients were using Triptans, 10 patients were using anti-inflammatory or analgesic agents, and 15 patients were using combinations. *P* min was found to be similar between migraine patients and controls. Although *P* WD and *P* max values of migraine patients were similar in migraine patients and healthy controls, the mean values were higher in migraine subjects as seen in Table 1. *P* WD was positively correlated with *P* max (*P* < 0.001). On the other hand, attacks number per month (*P* < 0.001) and male gender (*P* = 0.03) were the factors related to the *P* WD. In addition, *P* max was positively correlated with age (*P* < 0.05). VAS score was higher in females (*P* = 0.02). The presence of aura did not affect *P* value.

## 4. Discussion

Aura symptoms, gastrointestinal symptoms, and photosensitivity or phonosensitivity may be an imbalance of the sympathovagal imbalance in migraine patients [9, 15, 16]. In addition, supporting sympathetic dysfunction, the systolic blood pressure overshoot during the Valsalva maneuver was found to be decreased in migraineurs with aura [15]. Dysfunction of the ANS may affect atrial and ventricular repolarization. For example, increased sympathetic activity causes increased heart rate. Therefore, disrupted autonomic innervation of the heart and coronary arteries in patients with migraine may result in possible electrocardiographic (ECG) abnormalities during headache. Some case-control studies of cardiovascular function reported both sympathetic hyperfunction [16–18] and sympathetic hypofunction [19] even using single-lead ECG monitoring during migraine compared with a pain-free period.

Reduced parasympathetic and increased sympathetic nervous system (SNS) activity can lower the threshold for atrial fibrillation. Some previous studies have reported the association between the ANS and atrial fibrillation [20, 21]. But we have limited information on the association between atrial fibrillation and migraine. Duru et al. have shown that the migraine attacks were associated with increased QTc and *P*-wave dispersion compared with pain-free periods in a recent paper. Then, they concluded that patients with migraine during attacks were associated with increased QTc and *P*-wave dispersion because of a disrupted autonomic nervous system in migraine patients [13]. However, *P*-wave dispersion was not reported during pain-free period which might be a shower of damage related to attacks until now. In this study, we tried to find whether the patients with migraine may be under the risk of atrial and ventricular arrhythmias or not. For this reason, we undertook evaluation of *P*-wave dispersion as a sign of autonomic dysfunction in patients with well-defined migraine during headache-free period and compared to normal healthy controls. In our study, we found that *P* max and *P* min were similar between migraine patients and controls. Similarly, Aygun et al. reported that ECG abnormalities particularly PR and corrected QT (QTc) interval lengthening often were present during a migraine attack, and these abnormalities would be absent or less prominent during pain-free intervals [22]. Duru et al. also found that ECG abnormalities including *P*-wave dispersion were more prominent during migraine attack [13]. In addition to this previous knowledges we found that the mean value of *P* WD was higher in migraine subjects, and *P* WD was positively correlated with *P* max.

In conclusion, we believe that increased sympathetic activity may cause significant increase in *P* WD of migraine patients. The attacks number per month and male gender are the factors affecting the *P* WD, so the patients with higher numbers of attack may go under the risk of autonomic-dysfunction-related problems in the future.

## Figures and Tables

**Table 1:** Sociodemographical and clinical variables and *P*-wave durations in comparison.

Variable	Migraine group (*n* = 35)	Controls (*n* = 30)	*P* value
Age (year)	34.60 ± 7.54	35.87 ± 6.97	0.48
Gender			0.25
Male	6	9	
Female	29	21	
Number of the patients			
with aura	14		
without aura	21		
Duration of migraine (hour)	22.43 ± 22.85 (range: 3.0–72.0)		
Attack number per month	3.37 ± 1.63 (range: 2.0–8.0)		
VAS score	7.63 ± 2.11 (range: 4.0–10.0)		
*P* min value (ms)	41.81 ± 8.02	42.36 ± 8.07	0.78
*P* max value (ms)	95.97 ± 12.89	91.21 ± 10.05	0.11
*P* WD value (ms)	54.55 ± 13.44	50.40 ± 11.63	0.19
